# Evaluation of sonic, ultrasonic, and laser irrigation activation systems to eliminate bacteria from the dentinal tubules of the root canal system

**DOI:** 10.1590/1678-7757-2022-0199

**Published:** 2022-11-21

**Authors:** Chunhui Liu, Qiang Li, Lin Yue, Xiaoying Zou

**Affiliations:** 1 Peking University School and Hospital of Stomatology & National Center of Stomatology & National Clinical Research Center for Oral Diseases & National Engineering Research Center of Oral Biomaterials and Digital Medical Devices Department of Cariology Endodontology and Operative Dentistry Beijing China Peking University School and Hospital of Stomatology & National Center of Stomatology & National Clinical Research Center for Oral Diseases & National Engineering Research Center of Oral Biomaterials and Digital Medical Devices, Department of Cariology, Endodontology and Operative Dentistry, Beijing, China.; 2 Peking University School and Hospital of Stomatology & National Center of Stomatology & National Clinical Research Center for Oral Diseases & National Engineering Research Center of Oral Biomaterials and Digital Medical Devices Department of Emergency Beijing China Peking University School and Hospital of Stomatology & National Center of Stomatology & National Clinical Research Center for Oral Diseases & National Engineering Research Center of Oral Biomaterials and Digital Medical Devices, Department of Emergency, Beijing, China.

**Keywords:** Dentinal tubules, EDDY, Nd:YAP laser, Root canal disinfection

## Abstract

Aiming to kill bacteria in dentin tubules of infected dental pulp cavities, we evaluated the effects of sodium hypochlorite (NaOCl) solution agitated by different irrigation protocols, i.e., conventional needle irrigation (CNI), passive ultrasonic irrigation (PUI), the EDDY tip, and the neodymium-doped yttrium aluminum perovskite (Nd:YAP) laser. The EDDY achieved good antibacterial effects as passive ultrasonic irrigation in the coronal and middle thirds. Nd:YAP laser irradiation and PUI were effective in the apical third of the root canal.

**Objectives:**

To evaluate the ability of NaOCl agitated by high-frequency sonic irrigation–EDDY, PUI, and Nd:YAP laser–to kill bacteria in infected root canal walls and if the associated temperature increases at the root surface during application.

**Methodology:**

Infected root canal models were established, and roots were randomly divided into six groups: negative control, positive control, CNI, PUI, sonic agitation with EDDY, and Nd:YAP laser groups. After irrigation, the teeth were split and stained using the LIVE/DEAD BacLight Bacterial Viability Kit. Dead bacteria depth was evaluated by a confocal laser scanning microscopy and the temperature at the root surface was assessed using a thermal imaging camera during the irrigation process.

**Results:**

In the coronal and middle thirds of the root canal, PUI and EDDY had stronger antibacterial effects than CNI (p<0.05); in the apical third, the antibacterial effects of PUI and Nd:YAP laser-activated irrigation were better than CNI (p<0.05). The maximum change in temperature was significantly greater during continuous Nd:YAP laser application compared with the other methods, but intermittent irrigation helped lessening this trend.

**Conclusions:**

NaOCl agitated by EDDY tip and PUI exhibited a similar bacteria elimination effect in the coronal and middle root canal. Nd:YAP laser was effective in the apical third and intermittent irrigation reduced its thermal impact.

## Introduction

Chemo-mechanical cleaning and shaping of the root canal system to remove or reduce bacterial populations are essential for infection control, which is the main goal of root canal treatment.^1^ However, the complex anatomy of the root canal system limits the mechanical preparation, particularly in the apical third of the root.^2^ In infected root canals, bacteria can grow 400 μm or more into the dentinal tubules.^3^ Due to the strength and resistance of the remaining root canal wall, classic mechanical preparation generally only cuts the dentin at a depth of 150 μm, which is impossible to remove deep infection.^4^ Chemical irrigation is an effective supplemental method as it can reach more areas of the root canal surface.^5^ Techniques such as passive ultrasonic irrigation (PUI), sonic irrigation, and laser-activated irrigation enhance the disinfection effects of chemical irrigation and improve clinical outcomes.^6^

PUI activates irrigation via acoustic streaming and cavitation and its disinfecting effect is better than the conventional needle irrigation (CNI).^7,8^ The EDDY system (VDW, Munich, Germany) provides high-frequency sonic irrigation at 6000 Hz.^9^ Although previous studies have compared the antibacterial efficacies of EDDY, PUI, and CNI, the results have been inconsistent, presumably due to the different evaluation indicators employed.^10–12^ Most studies have quantified cultured residual bacteria, but some anaerobic bacteria are difficult to cultivate under normal conditions.^13^ Additionally, most studies^12,14,15^ used sterile paper points for sampling the root canals, however, this method only evaluates infection clearance for the overall root canal space, and not specifically for the dentinal tubules of the root canal wall.

Studies on the application of lasers to kill bacteria in root canals have been conducted since Fegan and Steiman^16^ (1995) evaluated the antibacterial effects of intracanal Nd:YAG laser irradiation, which may improve disinfection through a photothermal effect and by enhancing the chemical effects of irrigants.^17^

Commonly used lasers for root canal disinfection include the erbium-doped chromium-yttrium-scandium-gallium garnet (Er,Cr:YSGG) laser, the neodymium-doped yttrium aluminum garnet (Nd:YAG) laser, the neodymium-doped yttrium aluminum perovskite (Nd:YAP) laser, and the photon-induced photoacoustic streaming laser.^18^ Wang, et al.^17^ (2018) compared the antibacterial effects of different lasers on deep regions of dentinal tubules in a prepared dentin block infected by *Enterococcus faecalis*, resulting in Nd:YAP laser irradiation without NaOCl having the weakest effect among all. This finding probably relates to differences in the working tip construction. The end of the Nd:YAP laser fiber is flat, thus the light can only propagate in a straight line, which may result in bacterial survival. The Er:YAG and Er,Cr:YSGG lasers have conically shaped, radial firing tips that irradiate the root canals in three dimensions, leading to more widespread bacterial death.^19^ The Nd:YAP laser is a near-infrared laser with a wavelength of 1340 nm and working fiber diameter of 200–320 μm.^20^ Liu, et al.^21^ (2019) reported that Nd:YAP laser agitating NaOCl at 280 and 360 mJ achieved effective antibacterial effects. However, the study evaluated only the proportion of dead bacteria in the entire root canal wall. Najah, Sid, Ghodbane^22^ (2016) found that the antibacterial effects of 2.5% NaOCl agitated by Nd:YAP laser and passive ultrasonic irrigation were similar, in which both irrigation protocols showed better effects than CNI with 2.5% NaOCl. However, the bacterial counting method they used to compare the numbers of residual microorganisms failed to assess the dead bacteria deep in the dentinal tubules. We currently lack research evaluating the dead bacteria depth in the dentinal tubules of the root canal wall with Nd:YAP lasers, especially regarding the apical region. The main limitation of the laser method is its thermal impact during application, which may damage the periodontal ligament. Namour, et al.^20^ (2016) found that given proper working parameters, the temperature increase caused by the Nd:YAP laser during continuously endodontic irrigation with 2.25% NaOCl would not damage periodontal tissue. Moreover, Zhang and Wang^23^ (2021) reported that when using the Nd:YAP laser ablating separated files in root canal, the increase in temperature at the root surface was <10°C. But Rochd, Calas, Roques^24^ (1998) found that the temperature of the outer surface of the tooth root could increase by approximately 25°C after continuous laser operation in bacterial suspension for 28 s, which may cause thermal damage. Although clinicians usually use this laser intermittently for root canal disinfection, we lack research concerning the temperature increase.

Overall, few studies have focused on the effects of NaOCl agitated by the EDDY tip and Nd:YAP laser on eliminating bacteria in dentinal tubules of infected root canal walls, especially in the apical region. Most of the research concerning the thermal impact of Nd:YAP laser compares it to other lasers (or its outcomes using different parameters), and comparisons with PUI and sonic irrigation are lacking. Therefore, the aim of this study was to compare the antimicrobial efficiency of NaOCl agitated by the EDDY, PUI, and Nd:YAP laser with CNI. We hypothesized that neither EDDY nor Nd:YAP laser-activated irrigation would exhibit better antibacterial effects than PUI. This study also compared the temperature increase on the root outer surface caused by the Nd:YAP laser with ultrasonic, sonic, and syringe irrigation devices *in vitro*, thus providing experimental evidence for the selection of safe and effective final irrigation protocols in root canal treatment.

## Methodology

### Tooth selection and preparation

A total of thirty-eight mature single-root-canal premolars were collected from the clinic of the Department of Oral and Maxillofacial Surgery at Peking University School and Hospital of Stomatology. The study was approved by the institutional review board (approval No. PKUSSIRB-202058173). The sample size was determined using the program PASS for Microsoft Windows (ver. 15.0; NCSS Inc, Kaysville., UT, USA) and a randomized design.^25^ With a confidence coefficient of 0.95 (α=0.05) and a power of 0.9 (β=0.1), the minimum sample size for confocal laser scanning microscopy (CLSM) analysis was n=5 per group. Another ten teeth were used for the temperature increase experiment. To avoid any influence of root anatomy on temperature measurement, these teeth were reused. Only intact premolars with straight root canals were considered. The exclusion criteria were teeth with caries, periodontal defects, calcifications, apical resorption, or more than one canal, and root curvature >15°. The collected teeth were autoclaved at 121°C and 15 MPa for 20 min and stored in sterile water at 4°C for later use. Root canal preparation was conducted before bacterial infection according to a previously described protocol.^26^ Teeth were decoronated using a water-cooled high-speed bur (MANI, Tochigi, Japan) and observed under a dental microscope to confirm whether only one canal existed in each root. A #10 K-file (MANI) was inserted into the canal until the file tip was visualized at the apical foramen. Then, the roots were shortened to 12 mm via a K-file stopper. The working length (WL) of the root canal was set as 11mm, 1 mm shorter than the apical foramen. After removing the pulp tissue using a barbed broach (MANI), the root canals were prepared using ProTaper Universal instruments (Dentsply Maillefer, Baillagues, Switzerland), beginning with Sx and progressing to S1, S2, F1, F2, and F3. During root canal preparation, the canals were irrigated with 2 mL 5.25% NaOCl solution (Peking University School and Hospital of Stomatology, Beijing, China) for 1 min using a 27-gauge side-vented needle (Dentsply Tulsa Dental, Tulsa, OK, USA) within 2 mm from the WL after each instrument change. After the last instrument change, the canals were irrigated with 2 mL 5.25% NaOCl solution, 2 mL 17% ethylenediaminetetraacetic acid (EDTA; Peking University School and Hospital of Stomatology, Beijing, China) solution, and 2 mL sterilized water. The irrigation time for each solution was 1 min (1 mL/30 s). After preparation, the roots were autoclaved at 121°C and 15 MPa for 20 min and stored in sterile water at 4°C for later use.

### Establishment of *E. faecalis* infection

To confirm the cleanliness of the surface and exposure of dentinal tubules (which would allow bacterial incubation), two roots that had been subjected to root canal preparation were observed by scanning electron microscopy before the infection. The specimens were split in half using a chisel along the long axis and then fixed in 2.5% glutaraldehyde solution for 1 week. Thereafter, they were dehydrated in a graded series of ethanol solutions to a critical dried point, coated with gold, and examined via scanning electron microscopy (S2500; Hitachi, Tokyo, Japan). Each root canal was divided into apical, middle, and coronal thirds. A randomly selected location in the apical third was photographed at ×1,000 magnification, 5.0 kV. Then, two additional images were captured at 1 mm proximal and 1 mm distal to this site. Using the same protocol, three images of both the middle and coronal thirds were captured.

The infection protocol was like a published method.^27^ Briefly, a standard suspension (1×10^8^ cells/mL) of *E. faecalis* (ATCC29212; American Type Culture Collection, Manassas, VA, USA) was prepared in brain heart infusion medium (BHI) (Oxoid, Basingstoke, UK) at 37°C for 24 h. The *E. faecalis* suspension was filled to the root orifice level using a 27-gauge side-vented needle (Dentsply Tulsa Dental), showing a growth plateau after 12 h. The *E. faecalis* were cultured in 10 mL BHI broth at 37°C for 3 weeks and BHI medium was replaced every 48h to allow bacteria to grow into dentinal tubules. After incubation, the apical foramens were sealed with flowable composite (Ivoclar Vivadent, Schaan, Liechtenstein). Two additional roots were selected and observed via scanning electron microscopy to confirm that the infection deeply penetrated the dentinal tubules.

### Root canal irrigation protocols

After incubation, all roots were randomly divided into a negative control group (n=2), a positive control group (n=2), and four experimental groups (n=5 per group). In each experimental group, irrigation was performed using a distinct irrigation protocol. All canals were irrigated with 3 mL 5.25% NaOCl according to the following cycle: 30 s of 1 mL 5.25% NaOCl (1 mL/30 s), followed by 30 s of no irrigation. Then, 2 mL 17% EDTA (1 mL/30 s) and 2 mL sterilized water (1 mL/30 s) were delivered into the root canal and activated to remove residual irrigants.

#### Group 1: Negative control (n=2)

No root canal irrigation was performed in this group after bacterial incubation.

#### Group 2: Positive control (n=2)

Teeth were autoclaved at 121°C and 15 MPa for 20 min after bacterial incubation.

#### Group 3: CNI (n=5)

CNI was performed with a 27-gauge side-vented needle (Dentsply Tulsa Dental). Each canal was flushed with a continuous flow of 1 mL NaOCl for 30 s (1 mL/30 s) within 2 mm from the WL using a vertical motion and a 30-s soaking interval. This irrigating-soaking cycle was repeated twice (totaling three cycles). A total of 3 mL 5.25% NaOCl was used during this procedure. Then, 2 mL 17% EDTA was continuously flushed into the canal for 1 min (1 mL/30 s) within 2 mm from the WL with no soaking interval. Finally, 2 mL sterilized water was continuously flushed into the canal for 1 min (1 mL/30 s) within 2 mm from the WL, with no soaking interval. A rubber stopper was used to control the WL.

#### Group 4: PUI (n=5)

The canal was passively filled with 5.25% NaOCl. To irrigate, a 27-gauge side-vented irrigation needle was placed at the orifice level. The irrigant in the canal was activated using a PUI device (Satelec Acteon Group, Merignac, France) at the power setting of 7. A #25 ultrasonic file (Satelec Acteon Group) was placed 2 mm from the WL. Each canal was irrigated for 30 s with 1 mL 5.25% NaOCl (1 mL/30 s) using an ultrasonic device, followed by a 30-s soaking interval. This irrigating-soaking cycle was also repeated twice (total of three cycles). Then, 2 mL 17% EDTA was continuously flushed into the canal for 1 min (1 mL/30 s) within 2 mm of the WL under ultrasonic activation. Finally, 2 mL sterilized water.

#### Group 5: High-frequency sonic irrigation (n = 5)

The activation procedure in the high-frequency sonic irrigation group was like the PUI group. A #20 EDDY tip (VDW) was placed in the canal at 2 mm from the WL and operated in vertical motion.

#### Group 6: Nd:YAP laser (n=5)

A 200-μm Lokki Dt2 (Lobel Medical, Les Roches-de-Condrieu, France) tip was placed 2 mm from the WL after the canal had been filled with irrigant. The laser tip was operated at 280 mJ, 5 Hz, and 1.4 W with the intermittent irrigation protocol. First, the canal was passively filled with 5.25% NaOCl, as described above. The laser fiber was pulled up and down from the position 2 mm of the WL to the orifice level, and activated at 0–4 s, 13–17 s, and 26–30 s. No upward/downward movement or laser activation was performed, but only irrigation with 5.25% NaOCl solution was applied at 4–13 s and 17–26 s. A 27-gauge side-vented irrigation needle was placed at the orifice level to provide 1 mL 5.25% NaOCl during the 30-s (1 mL/30 s) intermittent irrigation. A 30-s soaking interval was also needed and this irrigating-soaking cycle was repeated twice (totaling three cycles). Then, 2 mL 17% EDTA was delivered continuously into the root canal for 1 min. Laser activation was performed twice according to the above mentioned 30 s intermittent irrigation protocol. Thereafter, 2 mL sterile water was irrigated according to the process.

### CLSM evaluation

After the irrigation, the roots were split in half longitudinally and stained using the LIVE/DEAD BacLight Bacterial Viability Kit (Molecular Probes, Inc., Eugene, OR, USA) for 15 min, according to the manufacturer's protocol. Then, the samples were observed via a CLSM device (LSM 710; Carl Zeiss, Oberkochen, Germany). For localization, each sample was observed at low magnification (×2). The apical, middle, and coronal thirds of the canal were defined 0–4, 4–8, and 8–11 mm, respectively, from the apical foramen, as the WL was 11 mm. The field of view was located near the longitudinal midpoint of each third, and three images of each sample were photographed at ×20 magnification. To observe live and dead bacteria, the wavelength was set at 480/500 nm for SYTO 9 (green fluorescent nucleic acid stain) and at 490/635 nm for propidium iodide (red fluorescent nucleic acid stain), respectively. Bacteria with intact cell membranes were stained fluorescent green, whereas bacteria with damaged membranes were stained fluorescent red. The width of red fluorescence at 300 μm was measured using ImageJ program (National Institutes of Health, Bethesda, MD, USA) and then used to calculate the depth of dead bacteria in each third of the canal. Each CLSM image was divided into 10 equal parts and the depth of red fluorescence was measured at the midpoint of each part. Data are expressed as mean ± standard deviation (μm).

### Heat production evaluation

Ten additional teeth were obtained and prepared as described above. The roots were irrigated using CNI, PUI, and EDDY with 1 mL 5.25% NaOCl for 30 s (1 mL/30 s), using the device parameters described above. The Nd:YAP laser was operated at 280 mJ, 5 Hz and 1.4 W with a 200-μm tip. Like the procedure described earlier, the canal was first passively filled with 5.25% NaOCl with constant irrigation. For the intermittent group, the laser fiber was pulled up and down from the apical third to the coronal third and activated at 0–4, 13–17, and 26–30 s. No upward/downward movement or laser activation was performed, but constant irrigation was applied with 5.25% NaOCl solution at 4–13 and 17–26 s. For the continuous irrigation group, the laser parameters were the same as for the intermittent group, though the activation procedure was applied continuously for 30 s, consuming 1 mL 5.25% NaOCl with no interruption. The roots were exposed to room temperature air and its outer surface temperature was measured over the entire 30-s period via a FORTRIC230 thermal imaging camera located 10 cm away from the roots. Via AnalyzIR program, the initial temperature of roots external surface and its highest temperature during the irrigation process were recorded. Then, the change in temperature was calculated. The same ten roots were used in each group to avoid errors caused by anatomical differences. For at least 30 min before reuse, roots were left to cool and maintain their initial temperatures.

### Statistical analysis

The normality of the data was assessed using a P-P plot. If the data had a normal distribution, one-way analysis of variance was used for the analysis, with the Games–Howell test applied for pairwise comparisons. For data not showing a normal distribution, the Kruskal–Wallis nonparametric test was applied. The significance level for all statistical analyses was set at α=0.05. Statistical analyses were performed via SPSS version 20.0 for Microsoft Windows (SPSS Inc, Chicago, IL, USA).

## Results

### Establishment of root canal models infected with *E. faecalis*

**Figure 1:**
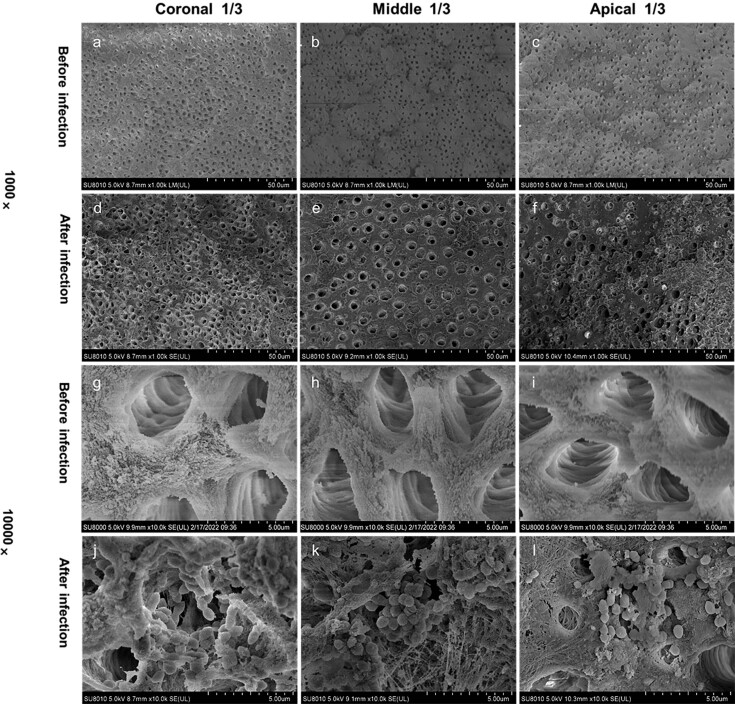
Scanning electron microscopy of the root canal wall before and after infection with *Enterococcus faecalis* (×1,000 and ×10,000); (a–c) the wall before infection under ×1,000 magnification; (d–f) the wall after infection under ×1,000 magnification; (g–i) open dentinal tubules without bacteria before infection under ×10,000 magnification; and (j–l) open dentinal tubules with bacteria growing after infection under ×10,000 magnification

Figure 1 shows representative images from the coronal, middle, and apical regions of each group. Before infection, the dentinal tubules of the canals were all open. After the 21-day infection process, the tubules were covered by bacterial colonies, confirming that the models had been successfully established.

### Dead bacteria depths of different irrigation protocols

**Figure 2:**
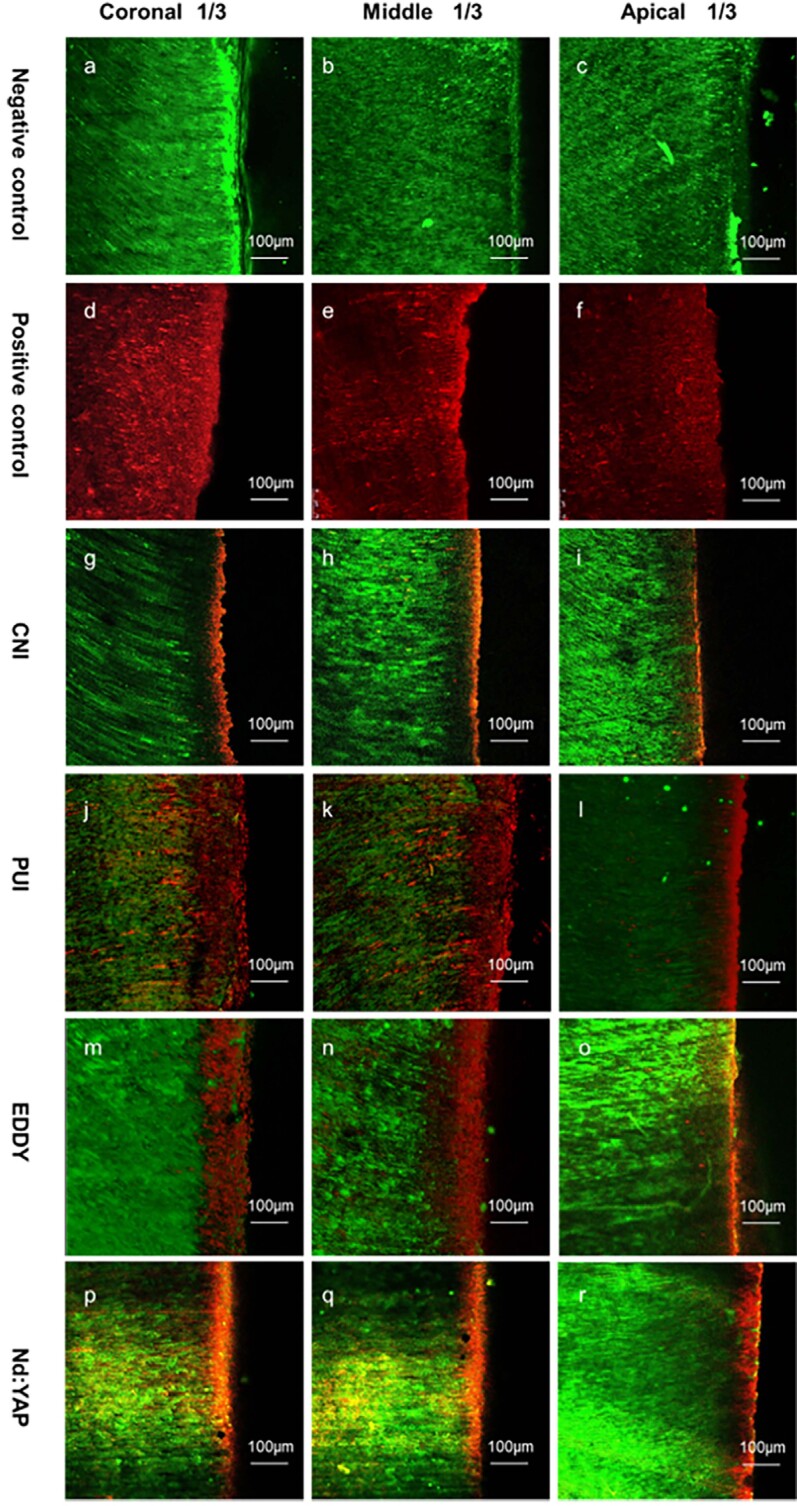
Distributions of live and dead bacteria in dentinal tubules. The black area is the root canal and the fluorescent area is dentin. The interface is the surface of the root canal wall; green fluorescence represents live bacteria and red fluorescence represents dead bacteria

**Figure 3:**
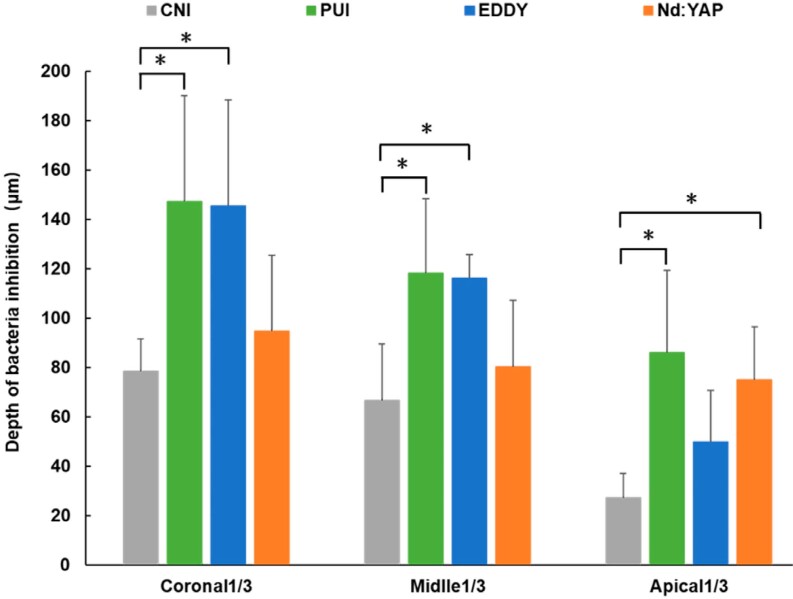
Quantitative analysis of dead bacteria in dentinal tubules. Graphs show the depths of dead bacteria of the dentinal tubules after the use of different irrigation protocols

Figure 2 shows representative images of live and dead bacteria distributions. The negative control group showed only green fluorescence (live bacteria) in all three fields of view with no red fluorescence (dead bacteria) in the dentinal tubules, indicating that nearly all bacteria remained alive. On the other hand, the positive control group showed only red fluorescence in all three fields of view, indicating that nearly all bacteria were dead. We observed several depths of red fluorescence for the test groups. Figure 3 shows the results of our quantitative analysis of dead bacteria.

The red fluorescence depths of the CNI, PUI, and EDDY groups showed a decreasing trend from the coronal to the apical third and we observed significant differences between the coronal and apical third in all three groups (p<0.05). The Nd:YAP laser group showed similar red fluorescence depths in the coronal, middle, and apical thirds (p>0.05).

In the coronal third, the red fluorescence depth in the PUI group (147.14±42.89 μm) preceded the high-frequency sonic irrigation group (145.38±39.96 μm), with no significant difference observed between groups (p>0.05). Both depths significantly differed from the CNI group depth (78.51±13.12 μm, p<0.05). The red fluorescence depth in Nd:YAP laser group was 94.80±13.28 μm, which was not significantly different compared to the PUI, EDDY, and CNI groups (p>0.05). We observed the same trends in the middle third. However, in apical third, the laser and PUI groups had good dead bacteria depth (74.93±8.08 μm vs. 86.12±33.18 μm, p>0.05). These were significantly better than the CNI group depth (27.34±9.73 μm, p<0.05). The bacterial killing effects of high-frequency sonic irrigation group in apical third was similar (49.94±20.72 μm, p>0.05) to the Nd:YAP laser, PUI, and CNI groups.

### Evaluation of heat production

All samples began at the same temperature (Table 1). Except for the CNI group, the temperature increased during the procedure in all of them.

**Table 1:** Temperature change at the root surface in each group

Group	Initial temperature (°C)	Maximum temperature (°C)
	(mean ± SD)	(mean ± SD)
CNI	26.75 ± 0.28^a^	27.33 ± 0.39^a^
PUI	26.46 ± 0.74^a^	33.00 ± 3.9^b,c^
EDDY	26.71 ± 0.89^a^	30.49 ± 2.90^b^
Nd:YAP continuous	26.83 ± 0.43^a^	55.89 ± 4.57^d^
Nd:YAP intermittent	26.72 ± 0.33^a^	35.00 ± 3.35^c^

SD, standard deviation.

The same superscript letter in a particular column indicates differences that are not statistically significant (p>0.05) according to one-way analysis of variance.

The mean increase in temperature failed to significantly differ between the EDDY (3.78±2.83°C) and PUI groups (6.53±3.59°C). Between the two laser-assisted irrigation groups, the intermittent irrigation group (8.28±3.45 °C) showed significantly smaller mean increase than continuous irrigation group (29.06±4.45°C). The intermittent group showed a significantly greater mean increase than the EDDY group. Overall, the continuous group showed a significantly greater mean increase than the other three groups. Thus, the laser groups were more likely to cause an increase in temperature than ultrasonic and sonic irrigation. However, intermittent irrigation may help to reduce thermal damage caused by the Nd:YAP laser.

**Figure 4:**
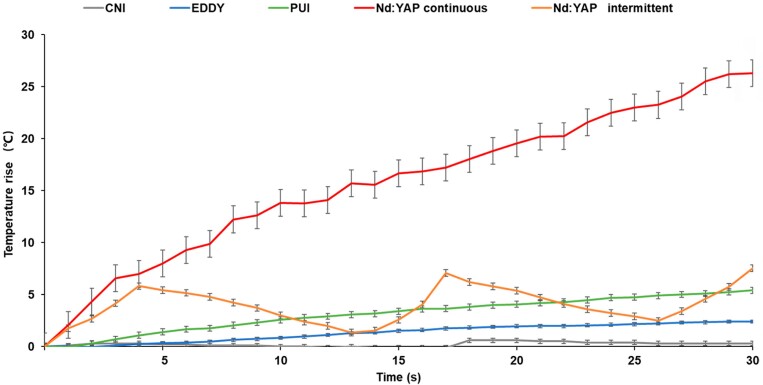
Increase in temperature at the root surface during 30 s of irrigation

Figure 4 shows the changes in temperature of each group recorded with 1-s intervals during the 30 s irrigation procedure. In the EDDY, PUI, and continuous laser groups, the temperature increased gradually over time. In the intermittent laser group, it increased at 0–4 s, 13–17 s, and 26–30 s, and decreased at 4–13 s and 17–26 s. The increase in temperature in the continuous irrigation group exceeded 10°C after 7 s and remained below 10°C in all other groups.

## Discussion

This study compared the antibacterial effects of NaOCl agitated by four irrigation protocols on infected root canal walls. We calculated the depth of dead bacteria in the dentinal tubules of coronal, middle, and apical segments of the root via CLSM images analysis. The data shows the efficacies of different irrigation protocols for removing infections in the dentinal tubules.

Several studies showed that the cleaning effects of dynamic irrigation are limited in the apical region.^12,27,28^ In our study, we found that the effects of NaOCl agitated by the PUI and EDDY showed a similar trend in the apical third. This finding probably relates to the small diameter of the root canal in the apical area and the short distance between the working tip and the root canal wall, which limits the effects of acoustic flow.^6^ However, we failed to find significant differences in the effects of the Nd:YAP laser in the coronal, middle, or apical thirds. The laser irradiation mainly inhibits bacterial growth via photothermal action. Its fiber head heat output can directly destroy the cell wall and the irrigants can absorb it, transferring heat into the dentinal tubules to kill bacteria.^18^ In this study, we moved the Nd:YAP laser fiber up and down 2 mm short of the WL, so that the irrigants in the root canal space could evenly absorb the heat. The uniform distribution of heat probably led to the relatively even depth of red fluorescence observed throughout the root canal.

In coronal and middle thirds, the antibacterial effect of the EDDY was equivalent to PUI. This finding indicates that the ultrasonic oscillation and sonic working tips could potentially achieve greater acoustic flow due to the unlimited space in middle and coronal parts of root canal.^29^ Although the EDDY frequency is lower than PUI, its vibration amplitude is larger. The irrigants velocity positively relates to the working tips amplitude^30^ Salas, et al.^31^ (2021) found that the penetration depth of 2% chlorhexidine irrigated by EDDY or PUI was similar in the cervical and middle region of the root canal. Meanwhile, the laser-activated irrigation was not significantly weaker than PUI or EDDY, nor significantly better than CNI. With CNI irrigation, irrigants have sufficient backflow space and the procedure is less affected by factors such as needle entry depth.^32^ These results suggest that, in the middle and coronal regions of the root canal, dynamic irrigation can achieve good results, whereas Nd:YAP lacks obvious advantages. However, because we removed more resistance in coronal third than what is commonly performed in clinical practice, coronal resistance may slightly differ between this study and an actual clinical situation, which may allow PUI and EDDY vibration to occur under less constrained conditions for potentially better effects.

Meanwhile, in apical third, the Nd:YAP laser showed an effective antibacterial effect like PUI and both groups showed significantly better antibacterial effects compared to CNI. The flexible and small in diameter fiber of the Nd:YAP laser can enter the prepared apical root canal smoothly and kill bacteria through its photothermal effects.^13^ The EDDY was neither significantly weaker than PUI nor significantly better than CNI. Ahmad, et al.^33^ (1988) found that lateral displacement of the tip of an ultrasonic file can reach approximately 40 μm, less than the oscillation amplitude of sonic working tips. Hence, tips used in the EDDY are more likely to contact the root canal wall than those of PUI. Walmsley, Lumley, Laird^34^ (1989) found that when the movement of the sonic file is constrained, its sideway oscillation disappears and its movement pattern can be converted into pure longitudinal vibration; thus reducing the irrigants penetration depth and clearing infections inside the dentinal tubules.

In this study, we choose a single-species biofilm model *E. faecalis* to establish an infection model. This model has been used in many studies^35,36^ and is considered effective for evaluating root canal disinfection. However, multi-species bacterial biofilm has been used in recent studies of infected root canals. Hoedke, et al.^37^ (2018) inoculated the root canal with *E. faecalis*, *Streptococcus oralis* (*S. oralis*), and *Prevotella intermedia* (*P. intermedia*) to analyze the antibacterial effect of photodynamic therapy. Swimberghe, et al.^38^ (2021) found that, when grown in a multispecies biofilm, *E. faecalis* showed significantly less susceptibility to NaOCl than a monospecies biofilm. However, the cultivation conditions for the multi-species bacterial biofilm may be stricter. For further and future research, our goal is to establish a multi-species bacterial biofilm infection model.

Najah, Sid and Ghodbane^22^ (2016) used the bacterial count method to compare the reduction of bacterial load in root canals after PUI and Nd:YAP laser-activated irrigation. The results failed to show significant differences between the groups. Calculating the reduction in bacterial load is a better solution because it represents a quantitative measure. However, this method mainly detects bacteria suspended in the root canal space and is not used to detect bacterial biofilm on the root canal wall or in the dentinal tubules. It also requires bacterial sampling, inoculation, and incubation increasing the chances of introducing microorganisms.^39^ In our study, we evaluated the depth of dead bacteria via CLSM. This step can be performed directly after staining the samples, which may reduce the potential of bacterial contamination.^40^ We focused on killing the bacteria in dentinal tubules, as we think that the depth of dead bacteria better reflects the antibacterial effect.

This study also showed that intermittent laser irradiation significantly reduced its heat-generation effect. The main limitation of laser is its thermal impact during application, which may damage the periodontal ligament^41^ and may cause postoperative pain.^42^ In this study, the maximum increases in temperature in the sonic irrigation, PUI, and intermittent Nd:YAP groups were all <10°C during the 30 s irrigation interval, which agrees with previous results.^20^ However, the increase in temperature in the Nd:YAP continuous irrigation group exceeded 10°C within 6–8 s; and its highest temperature during irrigation reached approximately 55°C. These results indicate that the heat generated by the laser is absorbed by water molecules in the dentin and then transmitted to the root outer surface. Therefore, to avoid damage to periodontal tissue when irrigating root canals, the Nd:YAP laser should be limited to intermittent use with short pulses.

This study had some limitations. First, the sample size was relatively small; and to avoid anatomical differences between groups and validate the results more strongly, further research with larger samples is needed. Second, the roots of teeth were exposed directly to air, whereas in another study the teeth were in 37°C water bath condition to simulate human body temperature.^20^ Under water bath conditions, the temperature at the roots outer surface could only be measured at specific points using a thermocouple, thus it may not represent its highest temperature reached.

## Conclusions

The NaOCl solution agitated by the EDDY system showed a potent bacterial killing effect in the dentinal tubules of coronal and middle thirds of root canal wall, but this effect was limited in the apical third. The NaOCl solution agitated by the Nd:YAP laser showed no advantage in terms of killing bacteria in dentinal tubules of coronal and middle thirds of the root canal compared to the PUI or EDDY but achieved similar antibacterial effect to the PUI in the apical third. Under the conditions used in this study, the increase in temperature at the root surface caused by the intermittent irrigation protocol is safe for clinical application.
